# Natural selection on plasticity of thermal traits in a highly seasonal environment

**DOI:** 10.1111/eva.12702

**Published:** 2018-10-09

**Authors:** Leonardo D. Bacigalupe, Juan D. Gaitán‐Espitia, Aura M. Barria, Avia Gonzalez‐Mendez, Manuel Ruiz‐Aravena, Mark Trinder, Barry Sinervo

**Affiliations:** ^1^ Facultad de Ciencias, Instituto de Ciencias Ambientales y Evolutivas Universidad Austral de Chile Valdivia Chile; ^2^ The Swire Institute of Marine Science and School of Biological Sciences The University of Hong Kong Hong Kong China; ^3^ CSIRO Oceans and Atmosphere Hobart Tasmania Australia; ^4^ Departamento de Ecología and Center of Applied Ecology and Sustainability (CAPES), Facultad de Ciencias Biológicas Pontificia Universidad Católica de Chile Santiago Chile; ^5^ School of Natural Sciences, College of Sciences and Engineering University of Tasmania Hobart Tasmania Australia; ^6^ MacArthur Green Glasgow UK; ^7^ Department of Ecology and Evolutionary Biology University of California Santa Cruz California

**Keywords:** acclimation, amphibians, Atacama Desert, natural selection, physiological plasticity, *Pleurodema thaul*

## Abstract

For ectothermic species with broad geographical distributions, latitudinal/altitudinal variation in environmental temperatures (averages and extremes) is expected to shape the evolution of physiological tolerances and the acclimation capacity (i.e., degree of phenotypic plasticity) of natural populations. This can create geographical gradients of selection in which environments with greater thermal variability (e.g., seasonality) tend to favor individuals that maximize performance across a broader range of temperatures compared to more stable environments. Although thermal acclimation capacity plays a fundamental role in this context, it is unknown whether natural selection targets this trait in natural populations. Additionally, understanding whether and how selection acts on thermal physiological plasticity is also highly relevant to climate change and biological conservation. Here, we addressed such an important gap in our knowledge in the northernmost population of the four‐eyed frog, *Pleurodema thaul*. We measured plastic responses of critical thermal limits for activity, behavioral thermal preference, and thermal sensitivity of metabolism to acclimation at 10 and 20°C. We monitored survival during three separate recapture efforts and used mark‐recapture integrated into an information‐theoretic approach to evaluate the relationship between survivals as a function of the plasticity of thermal traits. Overall, we found no evidence that thermal acclimation in this population is being targeted by directional selection, although there might be signals of selection on individual traits. According to the most supported models, survival increased in individuals with higher tolerance to cold when cold‐acclimated, probably because daily low extremes are frequent during the cooler periods of the year. Furthermore, survival increased with body size. However, in both cases, the directional selection estimates were nonsignificant, and the constraints of our experimental design prevented us from evaluating more complex models (i.e., nonlinear selection).

## 1 INTRODUCTION

It is well known that environmental temperature (*T*_a_) is the abiotic factor with major influence in the evolution, ecology, and physiology of most of the biodiversity in the planet (Angilletta, 2009 and references therein). The effects of *T*_a_ are particularly relevant for ectotherms as their body temperature (*T*_b_) depends on *T*_a_ and therefore any change in *T*_a_ affects their fitness and performance (e.g., behavior, growth, reproduction, metabolism). This relationship between performance and body temperature has been described by a thermal performance curve (TPC) (Angilletta, 2009; Huey & Berrigan, 2001) which has often been used to describe the thermal ecology and evolution of ectotherms (Gilchrist, 1995; Huey & Kingsolver, 1989), their phenotypic plasticity (Schulte, Healy, & Fangue, 2011), and to predict their responses to climate change (Clusella‐Trullas, Blackburn, & Chown, 2011; Sinclair et al., 2016). The TPC is best captured by three parameters: a minimum critical temperature (CT_Min_), which represents *T*_b_ below which performance is minimum; a maximum critical temperature (CT_Max_), which represents *T*_b_ above which performance is also minimum; and an optimum temperature (*T*_Opt_), which represents *T*_b_ at which performance is maximum. Although it is generally thought that preferred temperatures (*T*_Pref_) of ectotherms should be coadapted with *T*_Opt_ (Angilletta, 2009; Gilchrist, 1995), this requires organisms to be perfect thermoregulators, which usually it is not the case. In general, *T*_Pref_ is close to *T*_Opt_, but it is often lower than *T*_Opt_ (Martin & Huey, 2008). Within species, most of the TPC parameters can exhibit geographical variation depending on the particular environmental context (e.g., local climate) and genetic background of populations (Gilchrist, 1996; Kingsolver, Izem, & Ragland, 2004; Latimer, Wilson, & Chenoweth, 2011). This geographical variation has the potential to create gradients of selection for TPCs across the species distribution (Kingsolver & Gomulkiewicz, 2003) shaping thermal sensitivities, tolerances, and thermal acclimation capacities (i.e., thermal plasticity) of local populations (Gaitán‐Espitia et al., 2014; Seebacher & Franklin, 2012). At the interspecies level, on the other hand, TPC parameters (e.g., CT_Max_) have been considered good predictors of species’ acclimatory ability, geographical range size, and potential to cope with climate change (Calosi, Bilton, & Spicer, 2008; Sinclair et al., 2016; Stillman, 2003).

Different climate‐related hypotheses have been proposed to explain how physiological tolerances, capacities, and their plasticity affect the distributional ranges of species (Bozinovic, Calosi, & Spicer, 2011). One of them, the climate variability hypothesis (CVH), offers a powerful conceptual framework to explore the interactions between environmental variability and physiological performance of ectotherms (Gaitán‐Espitia, Arias, Lardies, & Nespolo, 2013, e.g., Gaitán‐Espitia et al., 2014). The CVH predicts that organisms inhabiting more variable environments should have broader ranges of environmental tolerance and/or greater ability to adjust their physiological traits to changes in environmental conditions (i.e., physiological plasticity) that enable them to cope with the fluctuating environmental conditions such as seasonality (Gaitán‐Espitia, Villanueva, et al., 2017; Ghalambor, Huey, Martin, Tewksbury, & Wang, 2006). In agreement with this hypothesis, other theoretical models have explored the evolutionary mechanisms underlying local thermal adaptation across heterogeneous environments (e.g., generalist–specialist models). For instance, environmental heterogeneity should select for more broadly adapted individuals (Lynch & Gabriel, 1987), whereas more constant environments should favor thermal specialists with narrow performance breadth (Gilchrist, 1995). The mechanistic understanding of these conceptual frameworks has improved with recent studies showing how in thermally variable environments directional selection acts on TPC parameters favoring organisms that maximize performance across a broader range of temperatures (Logan, Cox, & Calsbeek, 2014) despite the ability of ectotherms to thermoregulate behaviorally (Buckley, Ehrenberger, & Angilletta, 2015). Notwithstanding this progress, whether natural selection targets thermal acclimation capacity (i.e., physiological plasticity) itself in natural populations remains unknown. This is particularly true for ectotherms, which have been recently indicated to have rather low plasticity on thermal tolerance traits (CT_Max_, CT_Min_) (Gunderson & Stillman, 2015), and thus, they will have to depend on behavioral or evolutionary adjustments to buffer projected extremes temperatures.

In addition to increasing mean temperatures, it is known that climate change is changing the frequency and intensity of extreme temperatures and events (Rahmstorf & Coumou, 2011; Vázquez, Gianoli, Morris, & Bozinovic, 2017; Wang & Dillon, 2014). This, in turn, suggests that both averages and variances will have an important impact on different performance‐related traits (Bartheld, Artacho, & Bacigalupe, 2017; e.g., Lardies, Arias, Poupin, & Bacigalupe, 2014; Vasseur et al., 2014). Nevertheless, we still do not know whether selection might also target traits as a function of those extremes. In this context, populations inhabiting highly seasonal environments characterized also by daily extreme temperatures provide a natural laboratory to evaluate the role of natural selection on the plasticity of critical thermal limits and preferences. We addressed such important gaps in our knowledge by measuring for the first time survival as a function of the plasticity of thermal critical temperatures (CT_Max_ and CT_Min_), preferred temperature (*T*_Pref_), and thermal sensitivity of metabolism (*Q*_10_; the magnitude of change in metabolic rate for a 10ºC change in body temperature) after acclimating individuals to 10 and 20**°**C in the northernmost population of the four‐eyed frog *Pleurodema thaul*. Given that survival is a difficult trait to measure directly in the field as any unobserved individual can be dead or alive albeit undetected (Williams, Nichols, & Conroy, 2002; Kéry & Schaub, 2012), we used a mark‐recapture approach to estimate survival probability taking into account the recapture probability.

**Figure 1:**
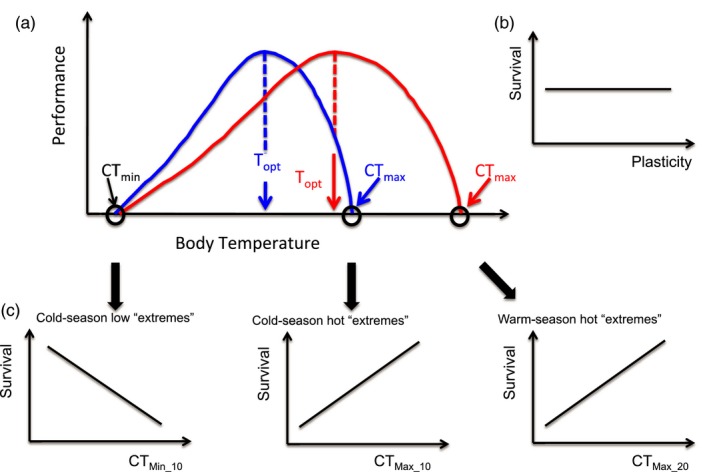
Graphical representation of the theory tested in this study. (a) Predictions developed from findings showing that acclimation to warmer temperatures produces an increase in the upper but not in the lower limits of the thermal performance curve (Ruiz‐Aravena et al., 2014). (b) The high seasonality should select for plasticity, and therefore, plasticity of all thermal traits should currently be under directional selection. (c) If daily low extremes are frequent, negative directional selection on CT_Min_ during the cooler periods of the year is expected (left panel). If daily high extreme temperatures are frequent, positive directional selection on CT_Max_ during the warmer periods (right panel) as well as the cooler periods of the year is expected (middle panel). We predict no directional selection on *T*_Pref_ and *Q*_10_ at both acclimation temperatures and on CT_Min_ when warm‐acclimated. Cold acclimation is indicated by a _10 subscript, while warm acclimation is indicated by a _20 subscript

We tested four predictions regarding phenotypic selection and plasticity that were derived from previous findings showing that acclimation to warmer temperatures produces an increase in the upper but not in the lower limits of the thermal performance curve (Ruiz‐Aravena et al.., 2014) (Figure 1). First, the high seasonality should select for plasticity in TPC parameters, and therefore, the plasticity itself should currently be under directional selection. Second, if daily high extreme temperatures were frequent, then we would expect positive directional selection on CT_max_ when warm as well as cold‐acclimated. Third, if daily low extremes were frequent, then we would expect negative directional selection on CT_min_ during the cooler periods of the year. Fourth, as energy inputs are limited, the energetic definition of fitness indicates that individuals with higher maintenance costs (i.e., resting metabolic rate) would have less energy available to allocate to growth, reproduction, and/or performance. The main prediction of this principle is that natural selection should maximize the residual available energy, and therefore, higher maintenance costs would be associated with lower fitness if no compensations in other functions occur (Artacho & Nespolo, 2009; Bacigalupe & Bozinovic, 2002). Thus, our final prediction is that *Q*_10_ is not under directional selection.

The understanding of whether and how selection acts on thermal physiological plasticity of natural populations is not just an important fundamental research topic in evolutionary ecology, but it is also relevant to other fields such as climate change and biological conservation (Chown et al.., 2010; Gaitán‐Espitia, Marshall, et al., 2017; Gaitán‐Espitia, Villanueva, et al., 2017; Merilä & Hendry, 2014). This is particularly true for populations in unpredictable, extreme, or heterogeneous habitats at the edge of the species distribution, because climate change is predicted to increase their risk of local extinction (Hoffmann & Sgrò, 2011). Under this context, traits such as thermal physiological plasticity will play a fundamental role determining the capacity and rate of acclimation of natural populations to anthropogenic global warming. Although there is some evidence suggesting that plasticity mediates some responses to climate change (Merilä & Hendry, 2014), to what extend the physiological plasticity itself is target of selection is unknown, and this makes our study unique. Selection can change across temporal and spatial scales, altering the capacity for thermal acclimation in populations and their resilience to climate change. Integrating this understanding into managing programs will improve planning conservation efforts aiming for the long‐term persistence of populations at the edges of species' ranges.

## 2 METHODS

### 2.1 Study organism and laboratory maintenance

Eighty‐three adult individuals of *P*. *thaul* were captured during September 2012 on two small ponds at Carrera Pinto (27º06′40.2′′ S, 69º53′44.3′′ W; 2,000 m.a.s.l.), a small oasis in the Atacama Desert that is known to be the northernmost population of the species (Correa, Sallaberry, Gonzalez, Soto, & Mendez, 2007). In both ponds, we performed an exhaustive search across microhabitats (below rocks, in the vegetation and in the water). All individuals were transported to the laboratory (Universidad Austral de Chile, Valdivia) within 2–3 days of capture. Following capture, all animals were marked by toe clipping and maintained in the laboratory for one month at a temperature of 20 ± 2°C and with a photoperiod 12D:12L. Animals were housed (*N* = 5) in terraria (length × width × height: 40 × 20 × 20 cm) provided with a cover of moss and vegetation and a small bowl filled with water. Individuals were fed once a week with mealworms (*Tenebrio sp*. larvae) and Mazuri® gel diets.

### 2.2 Acclimation and thermal traits

After one month at maintenance conditions, in a split cross design, half the frogs were acclimated to either 10 or 20**°**C for 2 weeks before measuring thermal traits. Frogs were randomly assigned to the first acclimation temperature using a coin. Next, they were acclimated to the other temperature, and again, thermal traits were measured. We chose these acclimation temperatures because they are close to the mean minimum temperatures during the breeding season (August–October, 10ºC) and to the mean temperatures during the active period of the species (20ºC) at Carrera Pinto ( http://www.cr2.cl). None of the investigators were blinded to the group allocation during the experiments. Body temperature of *P. thaul* reaches *T*_a_ within 90 min or less (Ruiz‐Aravena et al., 2014). This suggests that although terraria where they were maintained had moss and vegetation that might have been used in behavioral thermoregulation, the animals were completely exposed to 20 and 10**°**C for at least 15 days and thus fully acclimated to those temperatures.

Critical temperatures were determined as the environmental temperature at which an individual was unable to achieve an upright position within 1 min (Ruiz‐Aravena et al., 2014). Each individual was placed in a small chamber inside a thermo‐regulated bath (WRC‐P8, Daihan, Korea) at 30ºC (CT_Max_) or 5ºC (CT_Min_) for 15 min, after which the bath temperature was increased (or decreased) at a rate of 0.8ºC per minute (Rezende, Tejedo, & Santos, 2011). Every minute or at every 1ºC change, the chamber was turned upside down and we observed if the animal was able to return to the upright position. When an animal was unable to achieve an upright position within 1 min, it was allowed to recover at ambient temperature (CT_Min_) or for 30 min in a box with ice packs (CT_Max_). Body mass (a proxy of body size) was obtained before each trial using a Shimadzu TX323L electronic balance.

Preferred temperature (*T*_Pref_) was determined simultaneously for five individuals in five open‐top terraria (length x width x height: 85 × 12 × 30 cm). Each terrarium had a thermal gradient between 10 and 30ºC produced by an infrared lamp overhead (250 W) on one end and ice packs on the other. The organic gardening soil was moisturized at the beginning of each trial to prevent the desiccation of the frogs. Five individuals were placed at the center of each one of the terraria, and 45 min later, we registered *T*_Pref_ as the dorsal body temperature (*T*_b_) using a UEi INF155 Scout1 infrared thermometer. Body mass was obtained before each trial using a Shimadzu TX323L electronic balance.

SMR measured at 20 and 30ºC was estimated trough O_2_ consumption within an open system using a fuel‐cell O_2_ analyzer (FoxBox, Sable Systems, Las Vegas, Nevada, USA). A mass flow controller was used to supply 100 ml/min of dry CO_2_‐free air and a drierite and soda lime were used to scrub ambient air of water vapor and CO_2_. Frogs were placed individually in a cylindrical precision metabolic chamber (60 ml) covered with metal paper, and O_2_ consumption was registered over the course of 45 min per individual. The analyzer was calibrated periodically against a precision gas mixture. Although there was almost no difference between calibrations, baseline measurements were performed before and after each recording. Each record was automatically transformed by a macro program recorded in the ExpeData software (Sable Systems), to (a) transform the measure from % to mlO_2_/min, taking into account the flow rate and (b) to eliminate the first 5 min of recordings. For each individual, the metabolic sensitivity (*Q*_10_) was calculated as the ratio between metabolic rate measured at 30ºC and metabolic rate measured at 20ºC.

### 2.3 Selection on thermal traits

After the experiments, all frogs were put back to 20ºC for at least one month before releasing them. Marked frogs were released at Carrera Pinto in April 2013, and their survival was monitored on three separate recapture efforts (October 13, 2013, June 13, 2014, and September 9, 2014). As the desert surrounds these two small ponds, dispersal was not a concern. During each recapture event, two researchers sampled each pond exhaustively and every frog encountered was captured by hand while wearing a new pair of disposable nitrile gloves. Usually, in less than 24 hr, all visible frogs were captured.

The relationship between trait plasticity and survival was analyzed using the Cormack–Jolly–Seber (CJS) model, which is a class of open population capture–recapture models used specifically to estimate survival probability (Williams et al., 2002), that is, the probability that an individual in a given population survives from *t* to *t* + 1. In theory, survival probability can be easily estimated if we track the proportion of individuals in the population that die from *t* to *t* + 1 (Kéry & Schaub 2012). However, as the detectability of individuals in nature is almost always imperfect, we need to account for the observation process (i.e., we need to estimate a recapture probability) in order to get unbiased estimates of survival probability (Kéry & Schaub, 2012). The CJS model is the most widely used statistical model to jointly estimate recapture and survival probabilities in animal populations (Kéry & Schaub, 2012), and a review of its assumptions can be found in Williams et al. (2002). In our study, we first ran a goodness‐of‐fit test in the U‐Care 2.2 software (Choquet, Reboulet, Lebreton, Gimenez, & Pradel, 2005) to assess if our capture–recapture data were consistent with the assumed structure of the CJS model and to obtain a value for the over‐dispersion parameter (c‐hat). Subsequently, we fit the CJS model to the capture–recapture data using the Program MARK (Cooch & White, 2018). The structure of the CJS model was selected following a two‐stage process (Kéry & Royle 2016). First, based on AIC scores, we evaluated the best structure for recapture (constant, time‐dependent, and a linear trend) while keeping survival probability constant. Once the best structure for recapture was selected, we extended this model to evaluate the effect of the thermal traits on survival probability (see below). The time interval between capture occasions (as a fraction of 1 year and considering also the original capture event) was included in the analysis to accommodate the unequal time intervals. The resulting recapture and survival estimates were, therefore, corrected to annual estimates. A model selection and an information‐theoretic approach (Burnham & Anderson, 2003) were employed to contrast the adequacy of different working hypotheses (the candidate models) of selection on trait plasticity. The number of candidate models was kept to a minimum to minimize the likelihood of spurious results (Burnham & Anderson, 2003; Lukacs, Burnham, & Anderson, 2010). Body mass did not differ between acclimation treatments (*F*_1,174_ = 0.111, *p* = 0.74), and thus, average body mass was used in all subsequent analyses. Body mass showed a positive relationship with CT_Max_20_ (*r*_P_ = 0.47) and with *T*_Pref_10_ (*r*_P_ = 0.24) but was not associated with any other trait (results not shown). Therefore, we tested only for a null model (i.e., neither trait under selection), a model with body mass and models with directional selection for each trait separately and also for correlational selection (interaction of trait combinations) in the same trait at both acclimation temperatures, which indicates plasticity. Body mass was included as a covariate in the case of CT_Max_20_ and *T*_Pref_10_ (Table 1). All analyses were performed in R version 3.1.3 employing package RMark (Laake, 2013). No transformation was required to meet assumptions of statistical tests. Model parameters were obtained as the model averaged value across all candidate models weighted by individual model probability (Burnham & Anderson, 2003) (Table 1).

**Table 1:** Candidate models ordered accordingly to their Akaike weights

Models		*K*	AICc	ΔAICc	*w_i_*
1	Null model	2	130.17	0	0.220
2	CT_Min_10_	3	131.40	1.23	0.119
3	MB	3	131.78	1.61	0.098
4	*T*_Pref_20_	3	132.08	1.90	0.085
5	*Q*_10_10_	3	132.18	2.01	0.081
6	CT_Min_20_	3	132.25	2.08	0.078
7	CT_Max_10_	3	132.26	2.08	0.078
8	*Q*_10_20_	3	132.26	2.09	0.077
9	CT_Min_10_ + CT_Min_20_ + CT_Min_10_ * CT_Min_20_	5	133.38	3.21	0.044
10	MB + CT_Max_20_	4	133.44	3.27	0.043
11	MB + *T*_Pref_10_	4	133.82	3.64	0.036
12	*Q*_10_10_ + *Q*_10_20_ + *Q*_10_10 _* *Q*_10_20_	5	134.17	4.00	0.030
13	MB + *T*_Pref_10_ + *T*_Pref_20_ + *T*_Pref_10 _* *T*_Pref_20_	6	137.16	6.99	0.007
14	MB + CT_Max_10_ + CT_Max_20_ + CT_Max_10_ * CT_Max_20_	6	137.62	7.45	0.005

Note

AICc: AIC values corrected for small sample sizes; CT_Min_: minimum critical temperature; CT_Max_: maximum critical temperature; *K*: number of parameters; MB: body mass; *Q*_10_: thermal sensitivity of metabolism; *T*_Pref_: preferred temperature; *w_i_*: Akaike weights.

Single term models represent directional selection (e.g., CT_Max_), and correlational selection represents plasticity (e.g., CT_Max_10_ * CT_Max_20_).

Cold‐acclimated is indicated by a _10 subscript, while warm‐acclimated is indicated by a _20 subscript.

## 3 RESULTS

**Figure 2:**
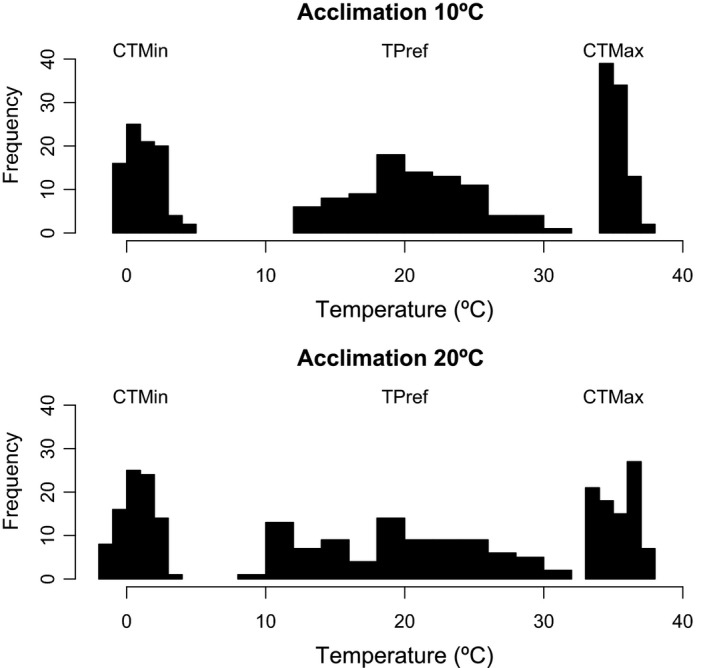
Frequency distribution of CT_Min_, *T*_Pref_, and CT_Max_ of the four‐eyed frog when acclimated to 10 and 20ºC

**Figure 3:**
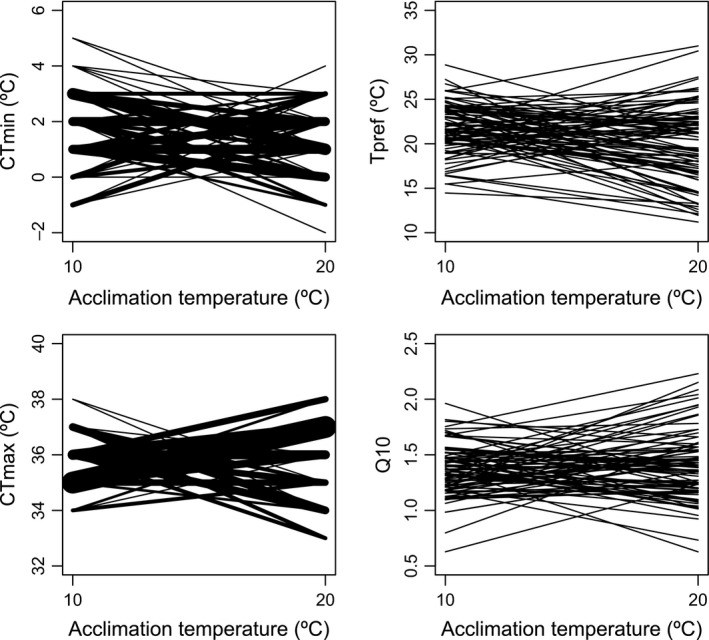
Individual plasticity in CT_Min_, *T*_Pref_, CT_Max_, and *Q*_10_ in response to 10 and 20°C acclimation treatments. Each line represents the individual value of the given trait at each acclimation temperature. For CT_Min_ and CT_Max_, the width of the line is directly proportional to the number of individuals that showed that specific response

All measured traits including critical thermal limits (CT_Max_, CT_Min_), thermal preference (*T*_Pref_), and sensitivity of metabolic rate to temperature (*Q*_10_) showed high variance among individuals (Figure 2). In addition, for all traits, some individuals shifted their thermal traits to higher values when acclimated to high temperatures, but other individuals showed the reverse response, that is, their traits shifted to lower values after acclimation at higher temperatures (Figure 3).

Only five out of 28 correlations between physiological traits were statistically significant, and these involved mostly critical thermal limits. In particular, CT_Max_20_ was negatively correlated with CT_Min_10_ (*r*_P_ = −0.57) and CT_Max_10_ (*r*_P_ = −0.41), while it was positively correlated with *Q*_10_20_ (*r*_P_ = 0.26). Additionally, CT_Max_10_ was positively correlated with CT_Min_10_ (*r*_P_ = 0.31) and negatively correlated with CT_Min_20_ (*r*_P_ = −0.25). The remaining correlations between traits were not significant (results not shown).

The overall goodness‐of‐fit measure for the CJS model indicated a moderate level of over‐dispersion (c‐hat = 2.65, *p* = 0.103); however, with only three recapture occasions, it was not possible to identify an alternative starting model, and the basic CJS model was adopted as the basis for subsequent model fitting, with unexplained over‐dispersion controlled using the c‐hat adjustment. A constant recapture rate was the best‐fit model irrespective of whether survival was modeled as a constant or time‐dependent rate (Table 1). Consequently, the constant rate‐recapture model was retained for subsequent modeling of survival. The model selection procedure indicated that from the 13 candidate models tested, there was not a single best‐fit one (Table 1). In particular, the null model was the most supported (Akaike weight of 0.220), while models including only directional selection on single traits still had some support, with a cumulative Akaike weight of almost 60% (Table 1). Models including correlational selection (i.e., plasticity) showed rather weak empirical support (Table 1). Overall, survival decreased as values of most of the traits increased in both warm‐ and cold‐acclimated conditions (Table 2).

**Table 2:** Directional selection estimates from single terms models with their standard errors (*SE*) and 95% confidence intervals (95% CI)

Trait	Estimate	*SE*	95% CI
MB	0.209	0.212	−0.206–0.625
CT_Min_10_	−0.248	0.187	−0.616–0.119
CT_Min_20_	−0.030	0.181	−0.384–0.324
*T*_Pref_10_	−0.025	0.059	−0.140–0.090
*T*_Pref_20_	−0.026	0.042	−0.109–0.056
CT_Max_10_	0.026	0.257	−0.477–0.530
CT_Max_20_	−0.192	0.195	−0.575–0.191
*Q*_10_10_	−0.475	1.140	−2.709–1.759
*Q*_10_20_	−0.048	0.795	−1.607–1.510

Note

CT_Min_: minimum critical temperature; CT_Max_: maximum critical temperature; *T*_Pref_: preferred temperature; *Q*_10_: thermal sensitivity of metabolism; MB: body mass.

Cold acclimation is indicated by a _10 subscript, while warm acclimation is indicated by a _20 subscript.

## 4 DISCUSSION

To understand how organisms adapt to highly fluctuating environments and whether they will be able to adaptively respond to current climate change, we need to evaluate whether selection in nature targets plasticity itself. Populations inhabiting highly seasonal environments that also experience daily extreme temperatures provide excellent opportunities to test predictions of the fitness consequences of such thermal variation on the plasticity of critical thermal limits and preferences. Here, to the best of our knowledge for the first time, we studied natural selection on thermal acclimation capacity of performance (CT_Max_ and CT_Min_), metabolism (*Q*_10_), and behavior (*T*_Pref_). Our results indicate that thermal acclimation in this population is not being targeted by directional selection, although there might be signals of selection on individual traits. In part, the relatively weak evidence for natural selection on this system might be a consequence of the small sample size we used (*N* = 88), the few recaptures we carried out (*n* = 3), and the relatively high value of c‐hat in the analyses, which penalizes models on the basis of parameter number. This prevented us not only from evaluating more complex models (i.e., nonlinear selection) but also resulted in estimates of directional selection with rather large SEs and therefore with 95% confidence intervals that contained the zero in all cases.

Some theoretical models of thermal adaptation across heterogeneous environments (e.g., climate variability hypothesis, generalist–specialist models) suggest that temporal environmental heterogeneity selects for more broadly adapted individuals (Gilchrist, 1995; Lynch & Gabriel, 1987), favoring increased plasticity particularly in thermal tolerance traits (Gunderson & Stillman, 2015). Based on these models, we predicted that the high seasonality should select for high plasticity in thermal traits, and therefore, the plasticity itself should currently be under directional selection. Our prediction turned out to be incorrect as models including plasticity showed relatively weak support.

Frogs of *P. tahul* in the Atacama Desert, the northernmost population of this species, are exposed to large daily and seasonal oscillations in environmental temperatures. The ratio between daily and annual thermal ranges (O'Donnell & Ignizio, 2012) experienced by this extreme population (0.65) is ca. 15% higher than that of a population 2,000 km south (0.52), which experiences narrower daily environmental temperatures at the center of the species’ distribution (Barria & Bacigalupe, 2017). This means that the studied population experiences a daily variation that is almost 65% of its seasonal variation. This high daily variation, in combination with the fact that climate change is already changing the frequency and intensity of extreme temperatures (Rahmstorf & Coumou, 2011; Vázquez et al., 2017; Wang & Dillon, 2014), made us wonder whether selection in nature might also target thermal traits as a function of daily extremes. As CT_min_ did not change through acclimation to warmer temperatures (Ruiz‐Aravena et al., 2014), we expected negative directional selection on CT_min_ during the cooler but not the warmer periods of the year. Our results are in agreement with the trend specified by this prediction, as survival decreased as CT_min_ increased (i.e., less tolerance to cold) when cold‐acclimated (albeit the estimate was nonsignificantly different from 0), which was the second most supported model (Table 1).

Although acclimation produced an increase in the upper limits of the thermal performance curve in this population (Ruiz‐Aravena et al., 2014), we expected positive directional selection on CT_max_ when warm as well as cold‐acclimated if daily high extreme temperatures were frequent. Our results do not offer support for this prediction: There was a slight trend for survival to decrease as CT_max_ increased under warm as well as under cold‐acclimated conditions. However, in both cases, estimates were not statistically different from zero. Nevertheless, this might suggest that selection could be favoring individuals that avoid hot microhabitats, possibly by means of behavioral responses (Ruiz‐Aravena et al., 2014). Indeed, behavioral thermoregulation has been proposed as one key factor that prevents an evolutionary response to selection to raising temperatures (Buckley et al., 2015; Huey et al.., 2012; Kearney, Shine, & Porter, 2009). The fact that CT_Max_20_ was negatively correlated with CT_Min_10_ indicates that individuals with higher cold tolerance might be the ones avoiding hot microhabitats, which opens very interesting questions for further research.

Regarding the sensitivity of metabolism to temperature (*Q*_10_), we expected that *Q*_10_ not to be under directional selection. Our results are in (partial) agreement with that expectation, as the rate at which survival changed with changes in *Q*_10_ was very small (Table 2), although the models with *Q*_10_ still showed some support (Table 1). Finally, we also expected no directional selection on *T*_Pref_ as we have previously shown that acclimation to warmer temperatures produced an increase in this trait (Ruiz‐Aravena et al., 2014). Nevertheless, we found a nonsignificant trend showing that survival decreased, although at a very low rate, as *T*_Pref_ increased, which might suggest that selection favors those individuals that are able to avoid hot microhabitats. It should be noted though that *T*_Pref_ was measured 45 min after an individual was put in the experimental terraria. As we were not in the experimental room during those 45, it is not possible to know whether an individual selected a specific temperature 1 min or 44 after being placed on the terraria, which might explain the huge phenotypic variation in this trait (Figure 1). Nevertheless, we do not consider this had any effect on the relationship between *T*_Pref_ and survival, as all evaluated models (Table 1) and not only those with *T*_Pref_ showed relatively weak evidence for natural selection.

Our results indicate a positive trend of survival with body size (the third most supported model, although the directional selection estimate was nonsignificant), something that has been previously reported in the literature (Aubin‐Horth, Ryan, Good, & Dodson, 2005; Crosby & Latta, 2013; Delaney & Warner, 2017; Iida & Fujisaki, 2007). This is somewhat unsurprising, given that body mass is known to be positively associated with several physiological traits that enhance performance (Castellano, Rosso, Doglio, & Giacoma, 1999; Hurlbert, Ballantyne, & Powell, 2008; Luna, Antenucci, & Bozinovic, 2009; Madsen & Shine, 2000; Shepherd, Prange, & Moczek, 2008) including plasticity itself (Whitman & Ananthakrishnan, 2009). Our oasis population inhabits two highly isolated ponds where other anuran competitors have not been observed, but there might be a risk of predation by herons (*L.D.B. personal observation*), which could explain the positive selection for body size. Nevertheless, further experimental work is needed to evaluate this possibility.

It is important to mention that we here measured plasticity in only one life stage. Likely, other ecological and physiological traits are also plastic in this species, and their responses to acclimation might differ, also among different life stages. Nevertheless, to persist in a warming world, evolutionary adaptation might be required when acclimatization responses reach their limit (Huey et al., 2012). As both the strength and shape of selection are key elements that impact the speed at which populations can evolve, determining whether selection in nature targets plasticity itself is of paramount importance. Our results show a signal and provide the first evidence that phenotypic plasticity is not an actual target of selection in nature, but that daily climate extremes might be selecting for higher tolerance. Nevertheless, further work including multiple traits and life stages and also in other populations should help to strengthen the trends found here into further generic hypotheses to clarify the role of plasticity for the viability of ectotherm populations in nature.

## CONFLICT OF INTERESTS

We declare we have no competing interests.

## AUTHOR CONTRIBUTIONS

L.D.B conceptualized the study, designed the experimental procedures, and carried out the experiment with A.M.B., A.G.M., M.R.A., and J.D.G.E. M.T., B.S., and L.D. B. analyzed the data and L.D.B., B.S., and J.D.G. wrote the paper with input from A.M.B and M.R.A.

## DATA ACCESSABILITY

Data are available for download from the CSIRO Data Access Portal ( https://data.csiro.au/dap/landingpage?pxml:id=csiro:29,733) https://doi.org/10.4225/08/5a9727318bd0f.

## ETHICS

This study did not involve endangered or protected species and was carried out in strict accordance with the recommendations in the Guide for the Care and Use of Laboratory Animals of the Comisión Nacional de Investigación Científica y Tecnológica de Chile (CONICYT). All experiments were conducted according to current Chilean law. The protocol was approved by the Committee on the Ethics of Animal Experiments of the Universidad Austral de Chile.
